# Tumor-associated neutrophils in renal cell carcinoma

**DOI:** 10.3389/fimmu.2026.1755401

**Published:** 2026-02-02

**Authors:** Olga V. Kovaleva, Vasiliy V. Sinyov, Madina A. Rashidova, Olga S. Malashenko, Alexei Gratchev

**Affiliations:** 1 Institute of Experimenal Oncology and Carcinogenesis, N.N. Blokhin National Medical Research Center of Oncology, Moscow, Russia; 2 Center for Molecular and Cell Biology, Skolkovo Institute of Science and Technology, Moscow, Russia

**Keywords:** inflammation, innate immunity, neutrophil, neutrophil extracellular traps, renal cell carcinoma

## Abstract

Renal cell carcinoma (RCC) is an immunogenic tumor in which tumor-associated neutrophils (TANs) and neutrophil extracellular traps (NETs) represent a functionally important component of the tumor microenvironment. Recent studies have revealed pronounced phenotypic heterogeneity of RCC-infiltrating neutrophils, including interferon-responsive, immunosuppressive PMN-MDSC-like, pro-angiogenic, and NET-forming subsets that cannot be adequately described by the classical N1/N2 model. Their polarization is shaped by ELR^+^ CXC chemokines (CXCL1, CXCL8), cytokine signals, systemic inflammation, hypoxia driven by VHL/HIF pathways, and tumor-intrinsic oncogenic alterations such as PTEN loss, ERβ- and c-Myc–dependent programs, as well as epigenetic remodeling. TANs exert predominantly pro-tumor functions in RCC, promoting T-cell exclusion and exhaustion, supporting angiogenesis and stromal remodeling, and facilitating epithelial–mesenchymal transition, venous invasion and metastasis. NETs, enriched in hypoxic and necrotic tumor regions and in venous tumor thrombi, further contribute to vascular occlusion, metastatic dissemination and local immune dysfunction, and are reflected by distinct transcriptional signatures. Clinically, high TAN density, activation markers and neutrophil/NET-associated gene signatures are consistently associated with aggressive tumor behavior, early recurrence, poor survival and resistance to VEGF-TKIs and immune checkpoint inhibitors. Emerging data also link neutrophil-rich stromal inflammation with the tumor resident microbiome, suggesting composite TAN-microbiome biomarkers for refined risk stratification. In this review, we summarize current knowledge on phenotypic diversity, regulatory circuits and functional programs of TANs and NETs in RCC, and discuss their prognostic and predictive significance, as well as therapeutic strategies aimed at chemokine blockade, complement modulation, NET inhibition and neutrophil re-education.

## 1 Introduction

Renal cell carcinoma (RCC) is an immunogenic tumor in which the tumor microenvironment (TME) strongly affects the disease progression and therapeutic efficacy. Despite significant improvement achieved with immune checkpoint inhibitors (ICIs) and ICI-TKI combinations, both primary and acquired resistance remain a frequent issue. Accumulating data implicate that granulocytic myeloid populations i.e. tumor-associated neutrophils (TANs), PMN-MDSCs, and neutrophil extracellular traps (NET)-forming neutrophils are active drivers of RCC progression, metastasis and treatment response (1, 2). Although these cells are consistently detected in RCC tissue, their functional states and clinical relevance are only beginning to be elucidated.

Neutrophils, long regarded solely as short-lived effectors, exhibit substantial phenotypic plasticity defined by cytokine gradients, metabolic stress, and stromal interactions. Within tumors, this diversity expands further, with acquisition of interferon-responsive, immunosuppressive (PMN-MDSC-like), pro-angiogenic, or NET-forming functional programs (3, 4). The classical N1/N2 model from murine experimental systems insufficiently describes this complexity. Single-cell RNA-sequencing in RCC supports pronounced neutrophil heterogeneity, though understanding TAN functional properties remains limited (4).

**Figure 1:**
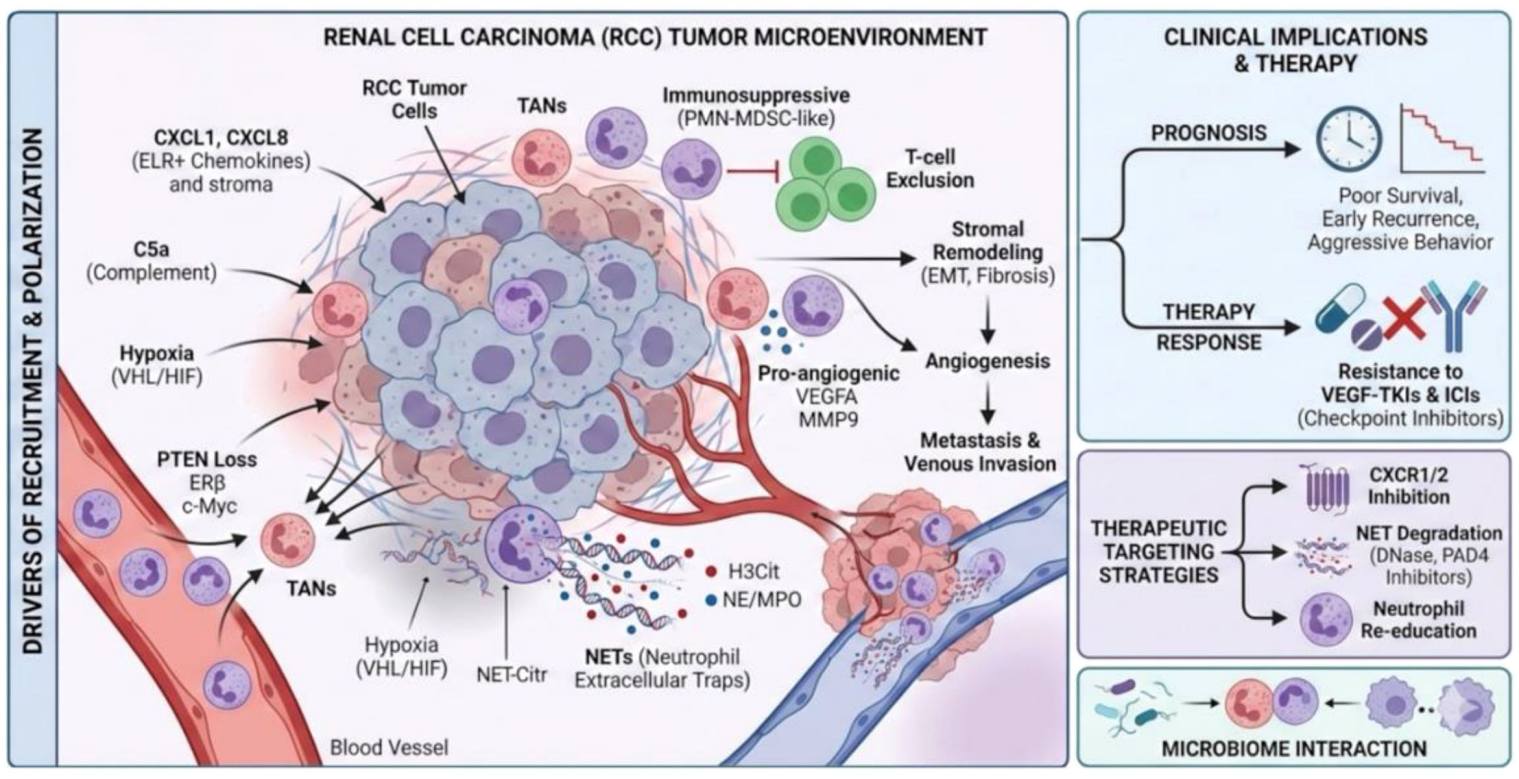
Tumor-associated neutrophils and NETs in the renal cell carcinoma microenvironment.

Multiple lines of evidence underline the importance of TANs and NETs in RCC. Elevated numbers of tumor-infiltrating neutrophils correlate with metastasis, EMT-like transcriptional programs, and poor prognosis in clear-cell RCC (2, 5, 6). Tumor-intrinsic alterations such as PTEN loss enhance CXCL1-mediated neutrophil recruitment, promoting immunosuppressive TMEs and ICI resistance (7). In parallel, NET markers including citrullinated histone H3 (H3cit) and myeloperoxidase (MPO) are increased in RCC relative to adjacent kidney and may stratify outcomes in metastatic disease receiving TKIs or combined treatments (8, 9). Collectively, these observations position TANs and NETs as integral components of RCC pathobiology with significant prognostic and therapeutic implications (**Figure 1**).

## 2 Phenotypic landscape of TANs in RCC

TANs are highly interesting and clinically relevant component of the immune microenvironment of RCC, with numerous studies linking their abundance with aggressive disease progression. Intratumoral CD66b^+^ neutrophils were early identified as an independent predictor of poor outcome in localized ccRCC (10), and this association extends to metastatic settings, for example in 271 mRCC patients, tumor infiltrating neutrophils predicted reduced survival in VEGF–TKI–treated but not cytokine-treated individuals, indicating therapy-specific prognostic implications (2, 6). Spatial profiling further refines these observations. Immunotherapy-exposed tumors show enrichment of stromal TANs aligned with collagen IV–integrin remodeling, suggesting that neutrophil localization marks resistant stromal niches (11). Accumulation of neutrophi elastase (NE)^+^ and H3Cit^+^ TANs, together with reduced epithelial E-cadherin, additionally links neutrophils to EMT-like programs and metastatic behavior of the tumor (12). Phenotypically, TANs in RCC demonstrates a broad spectrum of features that extends beyond N1/N2 models. Flow cytometry-based analyses demonstrate ARG1^+^, degranulation-associated, suppressive granulocytes - features further enhanced in obesity-associated tumors (13). Tissue studies also demonstrate increased neutrophil activation (MPO) in RCC tumor tissue compared with adjacent normal kidney tissue (14, 15).

Single-cell transcriptomic analysis revealed an expanded neutrophil state continuum in RCC. Six discrete neutrophil clusters (compared with two in healthy kidney) include inflammatory, interferon-responsive, catabolic, and autoimmune-like programs (4). Parallel analyses demonstrate that tumor-derived ELR^+^ CXC chemokines (CXCL8, CXCL1) correlate with accumulation of PMN-MDSC-like CD11b^+^CD15^+^HLA-DR^-^ granulocytes and higher tumor grade (3, 16), supporting chemokine-driven expansion of suppressive neutrophil populations.

Cross-tumor comparisons reveal both shared and RCC-specific features. While interferon-responsive, PMN-MDSC-like, and angiogenic neutrophil subsets are broadly conserved, RCC is distinguished by strong dependence on ELR^+^ CXCL-CXCR1/2 signaling (17–19), robust associations with EMT and necrosis (12), and stromal TAN enrichment following immunotherapy (11). Moreover, neutrophil-associated transcriptomic signatures stratify recurrence risk (20), integrating TAN biology into established prognostic frameworks.

## 3 Factors regulating TAN phenotypes in RCC

The phenotypic diversity of TANs in RCC is shaped by several factors, including tumor-intrinsic alterations, cytokine and chemokine signals, and local metabolic stress. Among these regulators, ELR^+^ CXC chemokines play a central role. Tumor-derived CXCL8/IL-8 promotes the expansion of PMN-MDSC-like CD11b^+^CD15^+^HLA-DR^-^ granulocytes, a population associated with higher tumor grade and increased risk of relapse (3), In parallel, loss of PTEN activates NF-κB/STAT pathways and increases CXCL1 production, leading to enhanced neutrophil recruitment and the formation of an immunosuppressive microenvironment that contributes to resistance to anti–PD-1 therapy (7). Together, these findings identify CXCL1 and CXCL8 as key drivers of TAN accumulation and suppressive polarization in RCC.

Cytokine-driven signals provide an additional layer of regulation for TANs in RCC. Although the classical N1/N2 concept originates from murine models, several features of RCC-infiltrating neutrophils resemble an N2-like, immunosuppressive phenotype. These cells commonly exhibit high ARG1 expression, signs of degranulation, and the capacity to inhibit T-cell activation. Systemic inflammatory conditions, particularly obesity-associated inflammation, further reinforce this suppressive programming. In obese patients, elevated levels of adipose-derived cytokines and metabolic mediators appear to shift TANs toward an ARG1-high, functionally inhibitory state, thereby amplifying immune suppression within the tumor microenvironment. Together, these observations highlight the sensitivity of TAN polarization to both local tumor-derived cues and broader systemic inflammatory signals (13).

Complement signaling is an additional regulator of TAN recruitment, polarization, and NET formation in RCC. Beyond pathogen defense, complement activation contributes to shaping the tumor microenvironment through C3/C5 cleavage products and signaling via C3aR/C5aR1, which can enhance neutrophil chemotaxis, activation, and immunosuppressive programs. In clear cell RCC, complement activation has been linked to adverse prognosis and to immune-contexture patterns relevant to immunotherapy, supporting its integration into TAN regulatory frameworks. Mechanistically, C5a-C5aR1 signaling is widely implicated in reinforcing suppressive myeloid circuits and may facilitate conditions permissive for NETosis in inflammatory microenvironments, suggesting convergence with ELR+ CXC chemokine-driven neutrophil recruitment in RCC (21–23). Recent mechanistic evidence further links RCC biology to complement activity, including tumor-derived extracellular vesicle–associated complement components that correlate with poor prognosis and can promote immune remodeling, supporting a direct tumor–complement axis rather than complement being only a bystander effect (24). These findings show that complement acts as an upstream amplifier, working with chemokine signaling and hypoxia-induced inflammation to direct TAN recruitment and polarization in RCC.

Hypoxia, driven by VHL loss and HIF stabilization, adds another layer of complexity to the regulation of neutrophil function. Hypoxic tumor regions accumulate elastase^+^ and H3Cit^+^ neutrophils, what indicates development toward NETosis (12). Therapy-exposed stromal areas with ECM remodeling also preferentially recruit neutrophils, suggesting that hypoxia-associated metabolic restructuring supports persistent TAN activation (11).

Tumor-intrinsic oncogenic pathways contribute as well. ERβ-dependent induction of VEGFA and HIF-2α enhances neutrophil-supported invasion (5), while androgen-driven c-Myc activity promotes neutrophil-mediated proliferation (25). Epigenetic mechanisms, including enhancer remodeling that facilitates neutrophil-dependent metastasis (6), further highlight tumor-directed programming of TAN states.

Taken together, these findings show that TAN phenotypes in RCC emerge from the combined influence of chemokine and cytokine signals, metabolic and hypoxic stress, and tumor-intrinsic oncogenic pathways. These converging factors drive the development of neutrophil subsets with distinct suppressive, inflammatory, and NET-forming capacities that contribute directly to RCC progression.

## 4 TAN functional programs in RCC

TANs in RCC engage a broad range of functional programs that affect tumor immunity, stromal organization, and overall tumor behavior. Although neutrophils may display anti-tumor activity in certain contexts, the available RCC data point predominantly to pro-tumor functions, including immunosuppression, support of angiogenesis, and contributions to tissue remodeling. These activities are further strengthened by tumor-intrinsic oncogenic pathways and the hypoxic conditions characteristic of RCC.

Multiple RCC studies demonstrate that TANs exert strong immunosuppressive activity within the tumor microenvironment. PMN-MDSC-like neutrophils expressing ARG1, degranulation signatures, and CD11b^+^CD15^+^HLA-DR^-^ phenotypes further contribute to local immune dysfunction and correlate with tumor grade and relapse (3). Tumor-intrinsic PTEN loss amplifies this suppressive environment by inducing CXCL1 and driving dense neutrophil recruitment, creating a T-cell-excluded state associated with diminished response to PD-1 blockade (7). Together, these mechanisms position TANs as important regulators of T-cell exclusion, exhaustion, and therapeutic resistance in RCC.

Beyond their immunosuppressive roles, RCC TANs engage chronic inflammatory programs that promote tumor progression. Tumor-derived signals stimulate TAN release of neutrophil elastase, matrix metalloproteinases, and reactive oxygen species, stimulating proliferation, migration, and immune escape. Mechanistic studies show that ERβ-driven VEGFA/HIF-2α signaling enhances neutrophil-mediated invasion (5), while androgen-driven c-Myc activation increases neutrophil-supported proliferative features (25). Although derived from cell-line systems, these findings illustrate how RCC-intrinsic pathways can engage neutrophil-driven inflammatory programs to support tumor progression.

TANs also contribute to angiogenesis and vascular remodeling. Through secretion of VEGFA, MMP9, and other pro-angiogenic mediators, neutrophils modulate vascular structure and enhance perfusion. Evidence from human tissues and experimental systems indicates that neutrophil-derived factors amplify canonical HIF-dependent angiogenic pathways, particularly in hypoxic and necrotic regions where TANs and NET markers accumulate (12). Recruitment via ELR^+^ CXC chemokines, especially CXCL8, promotes pro-angiogenic matrix remodeling and endothelial activation, linking TAN infiltration with vascular expansion (3).

Extensive crosstalk between TANs, the RCC stroma, and other myeloid populations further consolidates these tumor-supportive pathways. Spatial analyses indicate that neutrophils accumulate within ECM-rich stromal regions, particularly in immunotherapy-exposed tumors, where their distribution aligns with COL4A1-integrin remodeling (11). This localization suggests a contribution to stromal barriers that limit effective T-cell infiltration. TAN-derived proteases and cytokines modify the extracellular matrix, promoting increased stiffness, fibrosis, and angiogenic activity. Interactions with macrophages-mediated by shared chemokine circuits and overlapping immunosuppressive functions-likely strengthen the development of an immune-refractory tumor microenvironment.

## 5 Neutrophil extracellular traps in RCC

NETs represent a distinct effector mechanism of TANs and appear to have particular relevance in RCC (20). They consist of extracellular DNA decorated with citrullinated histones and granule proteins such as neutrophil elastase and MPO. Although classically associated with antimicrobial defense, NETs can also be triggered by tumor-derived inflammatory mediators, metabolic stress, and damage-associated signal (26, 27). The microenvironment of RCC is characterized by hypoxia, necrosis, and sustained inflammation and provides strong stimuli for NETosis (28). These conditions not only promote NET release but may also support the persistence of NET structures within the tumor tissue, thereby extending their potential impact on tumor progression, vascular remodeling, and local immune regulation. Complement activation can further potentiate NET formation in tumor tissues. Anaphylatoxins such as C5a act as potent neutrophil activators and may lower the activation threshold for NETosis under hypoxic and necrotic conditions typical of RCC. Given the emerging evidence for complement activation in aggressive RCC, complement–neutrophil crosstalk represents a plausible upstream layer reinforcing NET accumulation in perinecrotic zones and vascular invasion niches (21, 22).

### 5.1 NET formation mechanisms in RCC

In RCC, NETosis is closely associated with the metabolic and inflammatory conditions present within the tumor. Hypoxic and necrotic regions act as potent NET-inducing niches, as shown by the accumulation of H3Cit^+^NE^+^ neutrophils in perinecrotic areas (12). Tumor-derived CXCL8, a strong recruiter and activator of granulocytes, can further enhance degranulation programs that support NET formation (3). Tumor-intrinsic alterations, including PTEN loss, contribute additional signals by increasing CXCL1 production and promoting sustained neutrophil infiltration and priming (7). Although the detailed molecular pathways remain to be fully defined, current evidence indicates that local hypoxia, chemokine-driven recruitment, and oncogenic stress responses act together to promote NETosis in RCC.

### 5.2 NETs in RCC tissue, necrotic zones, and venous tumor thrombi

Histopathological studies consistently show that NETs are enriched within RCC tumors relative to adjacent kidney tissue. Using H3Cit and elastase immunostaining, Tessier-Cloutier et al. demonstrated that high intratumoral NET burden correlated with EMT-like changes and metastatic potential in 102 ccRCC cases (12). Complementary evidence from Liu et al. revealed increased neutrophil activation within tumor regions, supporting a NET-competent phenotype in RCC (15). NETs also appear prominently within venous tumor thrombi. Transcriptomic profiling of thrombus-associated neutrophils by Shang et al. identified a robust NET-related gene signature, differentiating thrombi from primary tumor tissue and providing molecular evidence that NETotic neutrophils contribute to thrombus biology in RCC (29).

These findings align with broader mechanistic principles: areas of vascular invasion and thrombosis are highly inflammatory, mechanically stressed, and hypoxic - all strong NET-inducing stimuli. The presence of NETs in thrombi suggests functional involvement in vascular occlusion, tumor cell anchoring, and metastatic dissemination.

### 5.3 Prognostic and predictive significance of NETs in RCC

NET-associated biomarkers demonstrate significant prognostic and potential predictive value in RCC. Increased NET burden in primary ccRCC, assessed by markers such as H3Cit, NE, and MPO, correlates with metastatic behavior, early recurrence, and reduced survival (12). In metastatic settings, transcriptomic signatures reflecting NET activity have been associated with aggressive tumor phenotypes and with therapy-resistant states, particularly in venous tumor thrombi (29). These observations indicate that NET formation is not only a histological feature but also reflects broader transcriptional programs linked to disease progression. Experimental data further suggest that NETs may hinder anti-tumor immunity by forming physical and biochemical barriers that restrict T-cell infiltration and function, although direct mechanistic evidence in RCC remains limited.

A recent NETosis- and TME-integrated prognostic model demonstrated strong predictive performance in clear cell RCC. By integrating NETosis-associated transcriptional programs with immune and stromal features in bulk and single-cell data, this framework stratified patients into risk categories with distinct survival outcomes and immune states, supporting the concept that NETosis is embedded within broader microenvironmental architectures rather than representing an isolated marker (30). Complementing these findings, other NETosis-related prognostic model studies in ccRCC similarly connect NETosis-associated programs with immune modulation and candidate immunotherapy implications (31).

At the transcriptomic level, several studies have identified neutrophil- and NET-associated gene signatures that stratify recurrence risk in localized ccRCC and reflect underlying tumor biology rather than isolated microenvironmental events (20). In addition, NET-focused molecular subtyping and signature approaches have been proposed for ccRCC, supporting NET-associated transcriptional programs as reproducible prognostic strata across datasets (32). These signatures often highlight coordinated inflammatory and stromal remodeling programs, reinforcing the concept that TAN- and NET-driven pathways contribute to clinically relevant tumor behavior.

Emerging RCC data also indicate that neutrophil-rich stromal inflammation is linked to the resident tumor microbiome. In a pilot cohort of 66 RCC patients, Kovaleva et al. combined 16S rRNA profiling with immunohistochemistry and demonstrated that intratumoral bacterial burden correlated with PU.1^+^ macrophages and CD66b^+^ neutrophils, and that, within tumors with high bacterial load, increased densities of these stromal cells were associated with poor prognosis (33). These findings suggest that combined assessment of tumor microbiome composition and neutrophil-dominated stromal inflammation could improve risk stratification and support the development of integrated TAN–microbiome prognostic markers in RCC.

## 6 Clinical and translational relevance

The growing body of RCC research highlights TANs and NETs as clinically informative components of the tumor microenvironment, with implications for prognosis, therapeutic response, and metastatic risk. Unlike circulating neutrophil-based metrics - which often lack tumor specificity - tissue-based TAN and NET assessments consistently correlate with clinically meaningful outcomes in RCC, particularly when spatial context and functional markers are incorporated.

### 6.1 Prognostic value of TANs and TAN-related signatures

Multiple studies demonstrate that TAN abundance predicts adverse outcomes in both localized and metastatic RCC. In early work, intratumoral CD66b^+^ neutrophils independently predicted poor survival in localized ccRCC (10). In metastatic settings, TIN positivity was associated with significantly shorter survival in VEGF-TKI-treated patients, but not in those receiving cytokine-based therapy, underscoring therapy-specific prognostic value (2). Supporting these findings, tissue analyses have revealed increased elastase^+^ and H3Cit^+^ TANs in aggressive tumors, linking neutrophil-rich microenvironments with EMT-like features, metastasis, and reduced survival (12).

Transcriptomic predictors further reinforce this prognostic association. A tissue-derived neutrophil signature identified by Ghatalia et al. accurately stratified recurrence risk in localized ccRCC, suggesting that neutrophil-associated transcriptional programs reflect deeper biological properties of tumor aggressiveness (20). These data collectively establish TANs, whether measured by density, activation markers, or transcriptomic surrogates as robust indicators of RCC progression.

### 6.2 Predictive relevance for targeted therapy and immunotherapy

TANs and NETs exert functional effects that influence therapeutic sensitivity. Neutrophil infiltration correlates strongly with resistance to targeted therapies. In metastatic patients receiving VEGF-TKIs, high TAN density predicted shorter progression-free and overall survival (2). Mechanistic studies further indicate that TANs can promote angiogenic and stromal remodeling programs that diminish TKI efficacy (5).

In the context of immunotherapy, tumor-intrinsic PTEN loss drives CXCL1-mediated neutrophil recruitment, generating an immunosuppressive microenvironment resistant to PD-1 blockade (7). Spatial profiling of post-immunotherapy RCC tissue reveals stromal neutrophil enrichment in ECM remodeling niches, suggesting that TANs may contribute to therapy-induced immune exclusion (11). Although clinical cohorts directly linking TAN states to ICI outcomes remain limited, available evidence supports TAN- and chemokine-driven suppression as contributors to immune escape.

NETs may also influence therapy response. NET-associated programs in venous tumor thrombi have been linked to aggressive phenotypes and transcriptional profiles associated with treatment resistance (29). Given their capacity to obstruct vessels, shield tumor cells, and exclude cytotoxic lymphocytes, NETs represent a mechanistically plausible mediator of therapeutic failure in RCC.

### 6.3 Association with metastasis and venous invasion

RCC is distinguished by its propensity for venous invasion and tumor thrombus formation—processes increasingly associated with neutrophil activity. High NET burden correlates with metastatic behavior and vascular invasion (12). Transcriptomic analyses of tumor thrombi demonstrate strong NET signatures that distinguish thrombus-associated neutrophils from those in primary tumor tissue (29). Coupled with reports of neutrophil-dependent metastatic processes driven by enhancer remodeling (6), these findings position TANs and NETs as active participants in the metastatic cascade rather than passive bystanders.

## 7 Therapeutic targeting of TANs

Although no TAN-targeted therapies are currently approved for RCC, several mechanistically grounded approaches are emerging from translational and preclinical research.

### 7.1 CXCR1/2 inhibition and chemokine blockade

The ELR^+^ CXC chemokines, particularly CXCL1 and CXCL8, play a central role in shaping neutrophil recruitment in RCC, making the CXCR1/2 axis an attractive therapeutic target. Preclinical models demonstrate that CXCR1/2 inhibition reduces neutrophil infiltration and attenuates the associated inflammatory and pro-angiogenic programs (17, 18). In PTEN-deficient tumors, where CXCL1-driven neutrophil recruitment contributes to resistance to PD-1 blockade, interrupting this pathway may help restore sensitivity to immunotherapy (7). Although clinical evaluation in RCC is still limited, these mechanistic insights provide a strong rationale for further investigation of CXCR1/2 antagonists, particularly in combination with immune checkpoint inhibitors.

### 7.2 NET-targeting strategies

Because NET burden correlates with aggressive behavior, venous invasion, and therapy resistance, targeting NETosis is an emerging translational objective. NET-associated signatures in tumor thrombi (29) and strong prognostic associations in primary tumors (12) suggest that PAD4 inhibitors, NE/MPO inhibitors, or DNase-based NET degradation may mitigate pro-metastatic and immunosuppressive effects. Although direct evidence in RCC models remains limited, NET inhibition has shown promising anti-tumor and anti-thrombotic effects in other malignancies, providing mechanistic rationale for RCC-specific evaluation.

### 7.3 Neutrophil re-education

Instead of depleting neutrophils, which risks compromising essential immune functions, current research is increasingly focused on strategies that modify their polarization. Tumor-intrinsic pathways such as ERβ-VEGFA/HIF-2α signaling (5) and c-Myc-driven proliferative programs (25) influence TAN behavior, suggesting that targeted inhibition of these axes may indirectly shift neutrophils toward less suppressive states. Modulation of metabolic or epigenetic mechanisms implicated in neutrophil-dependent metastasis (6) may further support the emergence of anti-tumor phenotypes. Although still in early development, these approaches reflect a broader movement toward immune reconditioning rather than myeloid depletion.

## 8 Conclusions and future directions

Neutrophils have emerged as key regulators of RCC progression, immune evasion, vascular invasion, and therapeutic resistance. Despite substantial advances, the functional relationships among TAN subsets and their coordinated interactions with stromal, endothelial, and lymphoid components of the tumor microenvironment remain incompletely defined. Meaningful progress will require integrated spatial-functional mapping of TAN states across primary tumors, venous thrombi, and metastatic sites, prospective validation of TAN- and NET-associated molecular signatures as prognostic and predictive biomarkers, and mechanistic studies capable of distinguishing context-dependent adaptations from lineage-intrinsic neutrophil programs. From a translational perspective, targeting ELR+ CXC chemokine circuits, complement–myeloid signaling, NETosis pathways, or neutrophil polarization represents a promising direction. As RCC therapy increasingly relies on immunomodulatory strategies, a refined understanding of TAN biology will be essential for designing interventions that reprogram, rather than deplete this highly plastic myeloid compartment.
